# Neurocognitive study of school performance among Moroccan high school students: The role of working memory

**DOI:** 10.1590/1980-57642018dn13-020013

**Published:** 2019

**Authors:** Aziz Eloirdi, Ahmed Ahami, Khaoula Mammad

**Affiliations:** 1 Team of Clinical Neuroscience, Cognitive and Health, Laboratory of Biology and Health, Faculty of Science, IbnTofail University, BP. 133, Kenitra, Morocco.

**Keywords:** neurocognitive, working memory, school performance, student, neurocognitivo, memória de trabalho, desempenho escolar, estudante

## Abstract

The Moroccan school system suffers from recurring dysfunctions as reported by the National Evaluation Body (NEB). These results lead to the degradation of learning and academic performance, in which several factors come into play. In Morocco, studies focusing on the neurocognitive profile of students and its influence on school performance are very limited or rare. In this context, the present study aims to study the role of working memory in school performance among Moroccan high school students.

**Methods:**

our sample contains 146 high school students. A total of 78 boys and 68 girls participated in this study and the numerical version of The Rey-Osterrieth Complex Figure test (ROCF) was used to assess working memory. Moreover, school performance is represented in this study by the mean obtained during the first semester.

**Results:**

the results of multiple linear regression revealed that working memory significantly explains variation in school performance.

**Conclusion:**

neuropsychological abilities, particularly working memory, significantly explain the deterioration in school performance of students reported by the National Evaluation Body.

## RESUMO.

O sistema escolar marroquino sofre de disfunções recorrentes, conforme relatado pelo *National Evaluation Body* (NEB). Esses resultados levam à degradação da aprendizagem e do desempenho acadêmico, que vários fatores entram em jogo. No Marrocos, pesquisas focadas no perfil neurocognitivo dos estudantes e sua influência no desempenho escolar são muito limitadas a raras. Nesse contexto, a presente pesquisa objetiva estudar o papel da memória de trabalho no desempenho escolar de estudantes do ensino médio marroquino.

**Métodos:**

nossa amostra contém 146 estudantes do ensino médio. Entre eles, 78 meninos e 68 meninas participaram deste estudo e utilizamos a versão numérica do teste de Rey-Osterrieth Complex Figure (ROCF) para avaliar a memória de trabalho. Além disso, o desempenho escolar representado neste estudo pela média obtida no primeiro semestre.

**Resultados:**

os resultados da regressão linear múltipla revelaram que a memória de trabalho explica significativamente a variação no desempenho escolar.

**Conclusão:**

as habilidades neuropsicológicas, particularmente a memória de trabalho, explicam significativamente a deterioração do desempenho escolar do estudante relatado pelo Órgão Nacional de Avaliação.

Based on the analytical report on the implementation of the National Charter for Education, Training and Scientific Research (NCETSR)(2000-2013), prepared by the Higher Council for Education, Training and Research Scientific (HCETSR) through the National Evaluation Body (NEB) in 2014,^1^ the Moroccan school system suffers from recurring dysfunctions. These problems have been noted in many national and international reports, such as the United Nations Organization for Education, Science and Culture(UNOESC) report in 2014,^2^ which classified the Moroccan education system as one of the weakest in the world. These dysfunctions, which concern the cohesion of the system and articulation of its components, the adaptation of training to meet demands of society, the integration of technologies and scientific research, all result in a lack of efficiency and performance of the Moroccan education system.

Indeed, performance ranks among the major dimensions of the education and training system that indicates its productivity. In fact, a school performance evaluation survey was conducted as part of the National Acquisitions Evaluation Program in 2008.^3^ According to this survey, 60% of students of the 6^th^ year of primary do not attain average marks. In high school, 92% of students of the 2^nd^ year and 84% of students of the 3^rd^ year score below average on mathematics. In physics and chemistry, 83% of the students of the 2^nd^ year and 86% of the 3^rd^ year obtained below average scores. In science of life and earth, these percentages exceed 90% for both levels. Regarding languages, such as Arabic and French, the results of the survey revealed that the majority of students failed to make the average. According to the NEB, the factors that lead to this degradation of achievements are the non-generalization of preschool, school delay, the teaching environment related to class size, the hourly load of teachers, the state of institutions, access to information, to communication technologies and the sociocultural environment of the family. However, these factors are all external, but there are internal factors related to the psychological and mental characteristics of the student that have an important role. Indeed, Fortin and Strayer^4^ believe that school success is the result of an interactive process between personal and environmental factors. In addition, Becker and Luthar^5^ suggest that youth mental health is one of the components of school performance. Moreover, studies focusing on the neurocognitive profile of adolescents and its impact on school performance are very limited in Morocco.

In this context, the present study aims to study school performance in terms of neurocognitive factors. More specifically, the role of working memory in school performance among Moroccan high school students was investigated.

## METHODS

### Participants

The study was conducted in December, January and February 2016 at the qualifying high school called “ZINEB NAFZAOUIA”, which is part of the province of Sidi Slimane of the Kenitra, Sale, and Rabat region, located in North Western Morocco. This is an exploratory descriptive study conducted with a sample of 146 students including 78 boys (mean age = 17.29 ± 1.40 years) and 68 girls (mean age = 16.94 ± 1.43 years). The participants were teenage students from both urban and rural backgrounds. Furthermore, the extracurricular and recreational activities are rare among the group and their socio-economic level generally seemed low.

In order to provide a representative sample of the general population, inclusion criteria and exclusion criteria were established.

- **Inclusion criteria:** students between 14 and 20 years old, attending a public educational institution, in a qualifying secondary education institution and studying all the subjects of programmed teaching in the qualifying secondary school.
- **Exclusion criteria:** uncorrected visual disorders, neurological antecedents (cerebrovascular accident or head trauma), in use of pharmacological treatment potentially causing attention disorders and/or drowsiness, severe depression or unstabilized psychiatric disorders.

### Tools used

#### The Rey-Osterrieth Complex Figure test (ROCF-A)

In order to evaluate working memory, the *ROCF-A*- digital version was employed, which consists of using:

- A digital pen (Anoto pen system) with a pencil lead and an infrared camera.
- Halftone paper with an usual appearance that does not disturb the writer, the frame consists of the juxtaposition of a myriad of points, a weft, generated by a mathematical algorithm. The pen reads the weft and is thereby precisely located.
- Expert Line Information Analyzer (ELIAN) software, developed by Seldage (http://www.eliansoftware.com) to visualize and analyze data from the pen. Several versions are available, we used the “Expert” version, which provides an Excel data table and an analysis to help the diagnosis. ELIAN provides access to very fine objective data (1/10 of mm, 1/100 of sec) and performs automated analyses. After making the layout, we put the pen in place, connected to the computer via its USB socket and ran the software “ELIAN”. The loading of the traces is done immediately.

The ROCF-A test is divided into two phases: for the copying phase, assessing visual perception, the model is presented horizontally to the subject and must be clearly visible. The subject is then asked to copy this drawing. The second phase of the test is reproducing from memory, assessing working memory: the model is subsequently removed and the subject asked to reproduce the figure again. The duration of the test is free of limits for both copying and reproduction. The two phases must be separated by an interval of less than three minutes.^6^ Two procedures are applied to the results: numerical scoring of points, which evaluates the quality of the production, and an analysis of the method of approach to the reproduction task.^7^

Thus, the numerical score makes it possible to establish a score, the FCR-A divided into 18 elements. These are listed one by one as follows: Correctly drawn and well placed (4 points), Correctly drawn and misplaced (2 points), Properly drawn, well placed but imperfect (3 points), Distorted or incomplete but recognizable and well placed (2 points), Distorted or incomplete but recognizable and misplaced (1 points) and finally Unrecognizable or absent (0 points). The type rating indicates the method or strategy used by the subject to copy and then reproduce the figure. To determine the type of organization used, the ELIAN software allows recording of the succession of features. According to Osterrieth,^8^ there are seven types of organization: 1) construction of the structure, 2) details encompassed in the structure, 3) general outline, 4) juxtaposition of details, 5) details on a confused background, 6) reduction to a familiar scheme, 7) scribbling. However, we were interested in the numerical rating.

#### Measure of school performance

School performance was represented in this study by the overall average obtained in the first semester of the 2015-2016 school year. This average was calculated from the results obtained for the different subjects programmed in the qualifying secondary school. The curriculum includes scientific subjects such as: Math, physics, chemistry, biology, and geology; Literary subjects such as languages, history, and philosophy; and finally, Physical education, and sports.

First, we studied the distribution of performance by gender, age, level of education and stream. Secondly, we studied this according to the working memory.

### Statistical analysis

The statistical analysis was based on two aspects: descriptive statistics and analytical statistics.

- **Descriptive statistics:** results for school performance, visual perception and working memory were expressed as mean ± standard deviation.
- **Analytical statistics:** Student’s *t* and ANOVA tests were used to compare the mean school performance by study groups, and correlation and regression tests were employed to assess the relationship between visual perception, working memory and school performance.

## RESULTS

### Distribution of the sample

Table 1 shows the distribution of the sample by gender, age, level of education, and stream. According to gender, boys constituted 53.4% of the population. For age, students under 17 represented 60.3%. Regarding level of study, the sample comprised 28.8% 1^st^ year high school, 39.7% 2^nd^ year and 31.5% 3^rd^ year high school students. Finally, for stream, scientists represented 45.9% of the sample.

**Table 1:** Distribution of the sample by gender, age, level of education and stream.

		Frequency	Percentage
Gender	Girls	68	46.6
	Boys	78	53.4
Age	≤ 17 years	88	60.3
	>17 years	58	39.7
Level of education	1^st^ year of high school	42	28.8
	2^nd^ year of high school	58	39.7
	3^rd^ year of high school	46	31.5
Stream	Scientist	79	45.9
	Literary	67	54.1

### Scores for visual perception and working memory

Table 2 presents the descriptive statistics of visual perception and working memory. The mean copying score (66.14) was higher than the reproduction score (40.79), and Student’s *t*-test confirmed that this difference was significant (t = 24.40, p < 0.001).

**Table 2:** Descriptive statistics of visual perception and working memory.

	Min	Max	Mean	SD
Score for copying (visual perception)	51	72	66.14	4.976
Score for reproduction (working memory)	10	70	40.79	12.225

### Study of school performance

In order to measure the normality of the distribution for school performance, the Kolmogorov-Smirnov test was applied and showed a normal distribution (p> 0.05).

The analysis of school performance (Table 3) showed a mean of 12.81 ± 1.92 (min = 8.74, max = 19.03). According to gender, the mean for girls was higher than for boys, where Student´s *t-*test showed that this difference was significant (t = 2.85, p = 0.005). This result implies that the girls learned better than the boys. In addition, for age, students aged under 17 years attained a higher mean than students aged over 17. This difference was statistically significant (t = 4.03, p < 0.001), where younger students performed better than older ones. With regard to level of study, there was a slight difference among the three levels. Analysis of variance showed that this difference was not significant (F = 0.19, p = 0.8). Finally, for stream, the mean for scientists was higher than that of literary workers. This difference was significant (t = 3.80, p < 0.001). This implies that scientists learned better than literary students.

**Table 3:** Means for school performance by gender, age, level of education and stream.

		Mean ± SD	Test	Sig
Gender	Girls	12.40 ± 1.86	t = 2.85	p = 0.005
	Boys	13.29 ± 1.88		
Age	≤ 17 years	13.31 ± 2.03	t = 4.03	P < 0.001
	>17 years	12.06 ± 1.44		
Level of education	1^st^ year of high school	12.88 ± 1.81	F = 0.19	p = 0.82
	2^nd^ year of high school	12.87 ± 2.15		
	3^rd^ year of high school	12.66 ± 1.72		
Stream	Scientist	13.34 ± 2.13	t = 3.80	p < 0.001
	Literary	12.18 ± 1.4		

Association among visual perception, working memory and school performance

Multiple linear regression was applied to seek a model that could possibly combine these three variables. In other words, one in which visual perception and working memory could predict the school performance of high school students.

Table 4, containing the correlations among the three variables, shows that visual perception and working memory significantly correlated with each other and with school performance.

**Table 4:** Correlation between school performance, visual perception and working memory.

	1	2	3
1. School performance (SP)	1		
2. Working memory (WM)	0.22**	1	
3. Visual perception (VP)	0.18*	0.13*	1

However, the multiple linear regression (stepwise method), having school performance as dependent variable and visual perception and working memory as explanatory variables, retained only working memory. Indeed, the standardized coefficient of the latter, which is 0.22, was found to be statistically significant (t = 2.73, p = 0.007), while that of visual perception did not significantly affect variation in school performance (t = 1.88, p = 0.06).

Therefore, the linear regression model is as follows:

SP=0.035WM+11.39

The model is statistically sound and expresses the data correctly (F = 7.44, p = 0.07).

## DISCUSSION

The objective of this study was to study the role of working memory in school performance among Moroccan high school students. To this end, 146 students participated in the study, classified according to gender, age, level of education and stream.

The results for visual perception and memory showed that mean copying score was higher than reproduction score. This result is consistent with results reported by Wallon and Mismin^6^ and by Alice.^9^ Indeed, although the rating system was the same for both phases, the reproduction phase requires additional skills.

In terms of school performance, the results of this study revealed that girls performed better than boys. Similar results were observed in a French survey on the education of girls and boys. Indeed, according to the survey, girls have better schooling paths than boys and are higher educated, where more girls hold Bachelor degrees.^10^ Therefore, in most developed OECD (Organization for Economic Co-operation and Development) countries, females are the more highly educated at the end of the secondary cycle compared to males. In addition, we have shown that the school performance of young adolescents is better than that of adolescents. This finding can be explained by the fact that young adolescents are, on the one hand, more dependent and under the control of their parents and teachers, whilst on the other, are less cognitively inhibited, allowing them to attain good results. In addition, this study has shown that school performance was better among scientist students. This difference may simply be due to the fact that the best students choose science subjects as a specialty.

The neurocognitive study of school performance showed that working memory significantly explains its variation. Indeed, it is the working memory that makes it possible to carry out cognitive tasks such as reasoning, reading and comprehension.^11^ In this sense, several studies have examined the relationship between working memory and school learning, which mainly concerns two aspects: 1) reading, the comprehension and writing of texts, and 2) resolution of operations or mathematics. Regarding the first aspect, to read and understand a text, it is necessary to relate perceptual and linguistic information with knowledge previously stored in memory, whereas the role of working memory lies in processing and storage for decoding and understanding a text.^12^ Thus, working memory allows the selection of appropriate information stored in long-term memory and its association with perceptual information.^13^ Many studies have shown that students’ reading performance is significantly associated with their working memory capacity.^14^^-^ 16 In addition, reading difficulties are often associated with a memory deficit,^17^ and students with a reading/learning disability have deficits in the executive functions supported by the central administrator.^18^ On the other hand, the relationship between working memory and written production has also been questioned. Indeed, the drafting of texts uses three mechanisms: formulation, execution and control.^12^ In fact, working memory is involved in the two processes of formulation, which are the planning of ideas and their translation into language (Kellogg, 1996) [18]. Thus, working memory is involved in the planning of ideas through the visuo-spatial component and in translation through the central administrator and phonological loop.^12^ Execution is managed by the central administrator.^19^ Finally, the control mechanism of improving texts during production uses working memory through the phonological loop and the central administrator.^12^

As for the operations resolution and mathematics, many studies have focused on the relationship between these types of task and working memory.^20^ Indeed, working memory is recruited as early as the first numerical activities performed by the child, such as enumeration^21^ and is involved in the most complex activities such as problem solving.^22^ Therefore, subjects with low working memory capacity commit more errors during enumeration activity^23^^,^ 24 and have difficulty writing dictated Arabic numbers.^25^ For the more complex mathematical operations, they use the phonological loop allowing encoding of numbers,^26^ the visuospatial component^27^ and the central administrator.^28^

In conclusion, at the end of this study, it emerged that neuropsychological abilities, particularly working memory, significantly explain the deterioration of school performance of students reported by the National Evaluation Body. Work that promotes the development of these skills, through more adapted learning situations, is necessary. This requires first training of teachers in the field of educational neurosciences. Further in-depth studies with brain imaging techniques could provide important insights in this direction.

In order to develop working memory at school, it is necessary to: Prioritize short instructions and short sentences; Rank information according to its importance; Prioritize the exercises or tasks to be done; Propose procedure assessment files; Be aware of the importance of the usefulness of the task; Explain these steps, the advances promoted by the task and splitting it up.
