# Erratum

**DOI:** 10.1590/1980-57642018dn13-020013erratum

**Published:** 2021

In the manuscript “Neurocognitive study of school performance among Moroccan high school students: The role of working memory”, DOI: 10.1590/1980-57642018dn13-020013, published in the Dement Neuropsychol. 2019;13(2):232-237, on page 232.

**Where it reads:**

Aziz Eloirdi^1^, Ahmed Ahami^1^, Khaoula Mammad^1^

^1^Team of Clinical Neuroscience, Cognitive and Health, Laboratory of Biology and Health, Faculty of Science, IbnTofail University, BP. 133, Kenitra, Morocco.

**It should read:**

Aziz Eloirdi^1^, Ahmed Ahami^2^, Khaoula Mammad^2^

^1^Team of Clinical Neuroscience, Cognitive and Health, Laboratory of Biology and Health, Faculty of Science, IbnTofail University, BP. 133, Kenitra, Morocco and the Hassan First University, Sports Science Institute, Settat, Marroco.

^2^Team of Clinical Neuroscience, Cognitive and Health, Laboratory of Biology and Health, Faculty of Science, IbnTofail University, BP. 133, Kenitra, Morocco.

